# Development and Evaluation of a MinION Full-Length 16S rDNA Sequencing Analysis Pipeline for Rapid Diagnosis of Animal Gastrointestinal Diseases

**DOI:** 10.3390/microorganisms13040777

**Published:** 2025-03-28

**Authors:** Ying Zhong, Qingyun Pan, Yu Wang, Jinyan Yu, Yaomen Li, Lifang Gu, Meicun Hou, Shenglong Liang, Jia Guo, Xinan Jiao, Yunzeng Zhang

**Affiliations:** 1 Jiangsu Co-Innovation Center for Prevention and Control of Important Animal Infectious Diseases and Zoonoses, Yangzhou University, Yangzhou 225009, China; m17863966079@163.com (Y.Z.); pqymmc@163.com (Q.P.); wy8042wy@163.com (Y.W.); eve_421@163.com (J.Y.); 17851375001@163.com (Y.L.); gulifang1997@163.com (L.G.); 15820873480@163.com (M.H.); 17851971057@163.com (S.L.); 2 Jiangsu Key Laboratory of Zoonosis, Yangzhou University, Yangzhou 225009, China; 3 Joint International Research Laboratory of Agriculture and Agri-Product Safety of the Ministry of Education, Yangzhou University, Yangzhou 225009, China; 4 Animal Hospital of Yangzhou University, Yangzhou University, Yangzhou 225009, China; 18068611426@163.com

**Keywords:** animal intestinal diseases, pathogenic bacteria infections, microbiota dysbiosis, MinION full-length 16S rDNA sequencing, bioinformatics analysis, rapid diagnosis

## Abstract

Rapid and accurate detection of the causes of gastrointestinal diseases in farmed and companion animals is crucial for advancing livestock farming and safeguarding public health safety. Diseases caused by pathogenic bacteria infections often result in the overrepresentation of pathogens in the gut microbiota; however, gut microbiota dysbiosis without obvious pathogen overrepresentation can also lead to disorders such as inflammatory bowel disease (IBD). Traditional cultivation-based diagnostic methods are time-consuming and ineffective in identifying microbiota dysbiosis-associated diseases. In this study, we developed a sample-to-answer MinION full-length 16S rDNA sequencing analysis pipeline, accompanied by detailed bioinformatics scripts, for the rapid diagnosis of animal gastrointestinal diseases. The pipeline enables the detection of pathogens and microbiota dysbiosis-associated diseases in approximately six hours. The pipeline showed high sensitivity and specificity, as evident by the analysis of artificially contaminated samples, and accurately diagnosed bacterial infections in five cases, including chicken, duckling, and piglet samples from their respective farms, as well as a companion cat, outperforming traditional methods. It also rapidly identified IBD in five companion animals. The findings highlight the potential application of our developed sample-to-answer analysis pipeline in pathogen detection and the diagnosis of gut microbiota dysbiosis-related diseases in animals, thereby improving livestock health and public safety.

## 1. Introduction

Pathogen infections are a major cause of diseases in animals and remain a significant concern due to their potential to cause epidemics both within and across species [1,2,3]. Particularly, diseases caused by pathogenic bacteria can have devastating effects. Epidemics triggered by pathogenic bacteria not only ravage the farming industry and lead to substantial economic losses [4,5,6,7], but they also pose a risk to human health, particularly through zoonotic transmission and the development of antimicrobial resistance [8,9].

The digestive tract serves as a key entry point for pathogenic bacteria, the disruptions in the host’s native microbiota by the pathogens can lead to disease onset [10]. For instance, *Mycobacterium avium* subsp. *paratuberculosis* infection can cause Johne’s disease in ruminants such as cattle and sheep [11,12], while pathogenic *Escherichia coli* colonization in the intestines of weaned piglets leads to diarrhea [13,14]. These gastrointestinal diseases cause significant economic losses in livestock breeding and production. For example, in the UK, Johne’s disease is estimated to cause an annual loss of USD 0.6 to USD 19 million to the cattle industry [15].

Given the shared living environments of companion animals and humans, pathogen infections in these animals can pose a public health risk if not detected promptly. Gastrointestinal diseases, which are highly prevalent in companion animals, often have complex etiologies, with a high risk of transmission to humans [16,17]. Of note, besides bacterial infections, gut microbiota dysbiosis-associated diseases, such as IBD, are also a main cause of gastrointestinal diseases in companion animals like dogs and cats [18].

In combating these diseases, the rapid and accurate detection of pathogenic bacteria is essential [19]. Traditional methods for detecting gastrointestinal pathogens largely rely on conventional cultivation techniques, which are time-consuming and often lack sensitivity, requiring days or even weeks for pathogen enrichment and detection [20,21,22]. Immunological assays, such as colloidal gold, and molecular biology assays, such as PCR, can only detect a limited subset of known pathogens and are unable to detect rare or novel pathogens. Furthermore, these methods do not identify gut microbiota dysbiosis-related diseases. Next-generation metagenomic sequencing (mNGS) has emerged as a valuable tool in clinical settings due to its ability to precisely detect pathogenic microorganisms accurately without the need for traditional cultivation, and it can also reveal the microbiota composition [23,24,25]. However, mNGS analysis is relatively time-consuming and requires specialized equipment, limiting its suitability for rapid on-site pathogen detection [26]. In contrast, MinION sequencing offers real-time, species-level resolution of the microbiota composition, allowing for rapid and accurate detection of pathogens, and has shown great promise in this area [27,28]. In particular, the MinION Mk1C device (Oxford Nanopore Technologies, Oxford, UK) is well-suited for on-site diagnosis due to its compact size, ease of use, and integrated analysis capabilities. Although the cost of MinION sequencing remains higher than traditional cultivation-based methods, it has become more affordable (less than 200 RMB per sample) and continues to decrease with advancements in sequencing technology. More importantly, this MinION sequencing-based approach significantly reduces the time required for clinical diagnosis. However, a comprehensive sample-to-answer pipeline for MinION full-length 16S rDNA sequencing and its application in diagnosing animal gastrointestinal disease is still lacking.

This study aims to establish a comprehensive pipeline encompassing sample collection, library construction, sequencing, and bioinformatics analysis for the detection of pathogenic bacteria and microbiota dysbiosis in livestock, poultry, and companion animals across various scenarios, utilizing MinION full-length 16S rDNA sequencing. The sensitivity and specificity of this pipeline were first assessed using simulated *Salmonella*-contaminated SPF (Specific Pathogen Free) pig fecal samples. The efficacy of the pipeline was further validated by diagnosing gastrointestinal pathogen infections and gut microbiota dysbiosis-related diseases in chickens, ducklings, pigs, dogs, and cats from clinical samples. Importantly, the entire process can be completed in approximately six hours, enabling the rapid detection of pathogenic bacteria and gut microbiota composition.

## 2. Materials and Methods

### 2.1. DNA Extraction

DNA was extracted from animal fecal samples using the QIAamp PowerFecal Pro DNA Kit (Qiagen, Hilden, Germany) following the manufacturer’s instructions.

### 2.2. MinION Full-Length 16S rDNA Library Preparation and Sequencing

The sequencing library was prepared using the SQK-RAB204 16S Barcoding Kit following the procedures described below. For each sample, the DNA concentration was diluted to 50–100 ng/μL, then full-length 16S rDNA PCRs were performed with 10 μL of DNA (total amount of 10 ng), 14 μL nuclease-free water, 1 μL 16S barcode at 10 μM, and 25 μL LongAmp Taq 2X master mix. If the DNA volume was less than 10 μL, nuclease-free water was added to compensate. The PCR cycling conditions were as follows: initial denaturation at 95 °C for 1 min, 25 cycles of denaturation at 95 °C for 20 s, annealing at 55 °C for 30 s, extension at 65 °C for 2 min, and final extension at 65 °C for 5 min.

Amplicons were purified using 30 µL of resuspended AMPure XP beads, followed by two washes with 200 µL of freshly prepared 70% ethanol, ensuring the pellet remained disturbed. After drying at room temperature, the DNA pellet was resuspended in 10 μL of 10 mM Tris-HCl pH 8.0 with 50 mM NaCl. DNA concentrations were measured with a Qubit 4.0 spectrophotometer, and 10 samples were pooled in equimolar ratios to reach approximately 100 ng of total DNA. The final sequencing library was prepared by adding 1 μL of RAP buffer, followed by 4.5 μL of nuclease-free water, 25.5 μL of loading beads (LB), and 34 μL of sequencing buffer (SQB). Finally, MinION nanopore sequencing was performed using R9.4 flow cells on a MinION MK1C device (Oxford Nanopore Technologies, Oxford, UK).

### 2.3. Bioinformatics Analysis

Real-time base-calling and sample de-multiplexing were performed using the Guppy algorithm (ver. 3.4.8) (https://community.nanoporetech.com, accessed on 15 January 2022) implemented in the MinION Mk1C release 20.03.5 software, and the reads with Q > 7 were kept for further analyses. The kept reads were further fed to Porechop (ver. 0.2.4) to trim the barcodes and adapters from the sequences [29]. Then, the reads shorter than 1000 bp or longer than 1600 bp were discarded using seqkit2 [30], and the remaining reads were fed to yacrd ver. 0.6.2 to remove chimeras in the samples [31]. Taxonomy annotations were assigned to the clean reads by mapping the reads to the rrnDB v 5.8 database using Minimap2 ver. 2 2.17-r941 [32], and only the alignments with the highest Smith–Waterman alignment score which was also higher than 1500 were reported [33].

### 2.4. Sensitivity and Specificity Assessment of the MinION Full-Length 16S rDNA Analysis Pipeline in Pathogen Detection Based on Salmonella-Contaminated Fecal Samples

A fecal sample of an SPF pig was collected under sterile conditions, and 10 g of the sample was transferred to a sterile sampling bag. The sample was thoroughly mixed and then separated into 12 aliquots. *Salmonella* Enteritidis strain C50041 was cultured in LB liquid medium and incubated at 37 °C with continuous shaking at 180 rpm for 12 h. The cells were harvested by centrifugation and resuspended in PBS to achieve a concentration of OD_600_ = 1. The *Salmonella* strain was introduced into the fecal samples at concentrations of 1 CFU/g, 10 CFU/g, and 100 CFU/g feces, ensuring thorough mixing in the sterile sampling bag, and a negative control group was also included. Each treatment was replicated three times to ensure robustness. Subsequently, DNA extraction was performed on these fecal samples, followed by MinION 16S rDNA library construction, sequencing, and bioinformatics analysis to evaluate the sensitivity and specificity of the pipeline. The absolute copy number of the total bacterial 16S rDNA gene in the fecal samples was determined using a qPCR method. A standard curve was generated by plotting the logarithm of the 16S rDNA copy number against the corresponding Ct values, resulting in a linear equation y = −3.953x + 44.78. The relative abundance of contaminated *Salmonella* in the microbiota of fecal samples was then calculated accordingly.

### 2.5. Clinical Sample Collection and Sequencing Analysis

During the time from December 2021 to March 2022, the fecal samples were collected using rectal swabs from patients with obvious symptoms of gastrointestinal diseases at the Animal Hospital of Yangzhou University. Six samples were from the pet clinic and four samples were from the livestock clinic. All samples were stored in dry bead tubes provided in the QIAamp PowerFecal Pro DNA Kit and immediately transferred to the Key Laboratory of Zoonosis of Yangzhou University on ice. The DNA was extracted and MinION full-length 16S rDNA sequencing analysis was performed to evaluate the efficacy of the developed pipeline in gastrointestinal disease diagnosis.

## 3. Results

### 3.1. Development of Sample-to-Answer MinION Full-Length 16S rDNA Sequencing Analysis Pipeline

**Figure 1:**
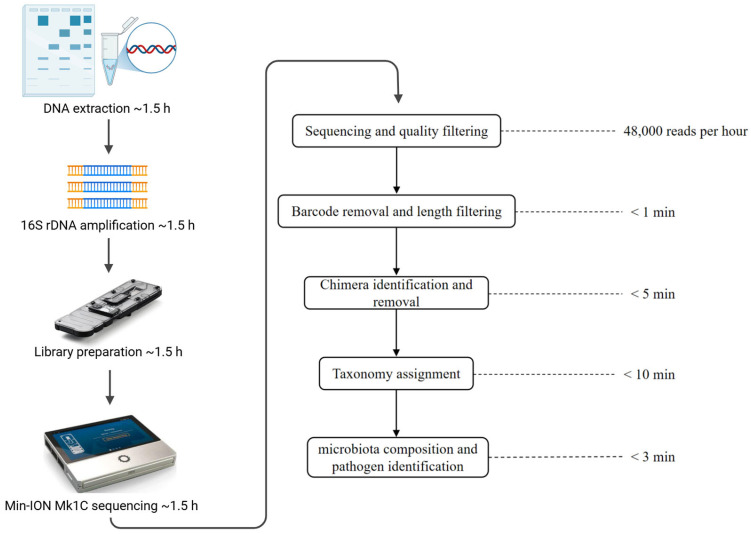
Overview of the developed MinION full-length 16S rDNA sequencing pipeline (Created with BioRender (https://www.biorender.com/), agreement No. VN282Q8FNP, accessed on 6 April 2024). The estimated time cost in each step is indicated.

The sample-to-answer time of our developed MinION full-length 16S rDNA sequencing pipeline for animal gastrointestinal disease diagnosis was approximately 6 h. This included ~1.5 h for DNA extraction, ~1.5 h for 16S rDNA amplification, ~1.5 h for MinION sequencing library preparation, and ~1.5 h for sequencing and corresponding bioinformatics analysis (Figure 1). Specifically, upon initiating on-machine sequencing with the MinION Mk1C sequencer in our laboratory, real-time base-calling and sample de-multiplexing were performed. Approximately 48,000 (quality score Q > 7) 16S rDNA reads were generated per hour (approximately 4000 reads per sample per hour when sequencing pooled DNA samples of 12 individuals). The generated reads were trimmed and filtered using an in-house script as follows (using a sample S1 as an example):

porechop -i S1.fastq|seqkit seq -m 1000 -M 1600 - > S1.1-1.6kb.fq

Only reads with lengths ranging from 1 to 1.6 kb were retained to ensure both sequence quality and relevance. This step was completed in under one minute.

Potential chimeric sequences were identified and discarded using the following commands:

minimap2 -x ava-ont -g 500 S1.1-1.6kb.fq S1.1-1.6kb.fq > S1.1-1.6kb.overlap.paf
  yacrd -i S1.1-1.6kb.overlap.paf -o S1.1-1.6kb.report.yacrd -c 4 -n 0.4 scrubb -i S1.1-1.6kb.fq -o S1.1-1.6kb.scrubb.fq

The runtime for this process was under five minutes.

Finally, the taxonomic affiliation of the reads was classified by aligning the reads to the rrnDB database, which houses over 28,000 full-length 16S rDNA sequences representing more than 7500 prokaryotic species. Only the best hits, with a mapping score (DP score) greater than 1500 as determined by minimap2 were kept for taxonomic classification. The command parameters were used:

minimap2 -a -x map-ont --secondary = no -t 46 rrnDB-16S_rRNA.name.fasta S1.1-1.6kb.scrubb.fq > S1.1-1.6kb.scrubb.rrn.sam
  cut -f1,3,14 S1.1-1.6kb.scrubb.rrn.sam |grep ‘AS:i’ |sed ‘s/AS:i://g’ |awk ‘{if ($3≥1500) print $0}’ > S1.1-1.6kb.result.txt

The step took less than ten minutes to complete.

The generated S1.1-1.6kb.result.txt file could be exported into an Excel spreadsheet, where the identified bacterial species were sorted in descending order based on the number of corresponding sequencing reads. This allows for facile identification and inquiry into the types and prevalence of pathogens as well as microbiota dysbiosis within the dataset. Reviewing and organizing these data in Excel can be manually accomplished within approximately 2–3 min.

In summary, the developed pipeline enables the rapid and accurate characterization of microbiota structure at the species level within approximately 6 h, facilitating the identification of potential pathogens or microbiota dysbiosis associated with animal gastrointestinal diseases.

### 3.2. Sensitivity and Specificity Assessment of the Developed Pipeline in Pathogen Detection Based on Salmonella-Contaminated Fecal Samples

To assess the sensitivity and specificity of the developed MinION full-length 16S rDNA sequencing pipeline for pathogen detection, a simulated contamination experiment was conducted. Briefly, an SPF pig fecal sample was thoroughly mixed and then divided into 12 aliquots. Different amounts of *Salmonella* cells (0, 1, 10, and 100 CFU/g fecal samples) were added into the aliquots, with each concentration of *Salmonella* repeated three times. DNA was extracted from these fecal samples, followed by MinION sequencing and bioinformatics analysis. No false positives for *Salmonella* were detected in the non-contaminated SPF fecal samples, confirming the high specificity of the bioinformatics analysis pipeline employed in this study. The results demonstrated the successful detection of *Salmonella* even at extremely low relative abundances (1.06 × 10^−7^) within the microbiota (Table S1), indicating the exceptional sensitivity of this pipeline. Thus, the pipeline enables precise pathogen identification even at exceedingly low levels, without the need for any enrichment procedures.

### 3.3. Evaluation of the Developed MinION Full-Length 16S rDNA Sequencing Pipeline in Animal Gastrointestinal Disease Diagnosis Practices

Fecal samples from sick animals, collected from the livestock, poultry, and pet clinics of the Animal Hospital of Yangzhou University, were analyzed using MinION full-length 16S rDNA sequencing to evaluate the efficiency of the developed pipeline for animal gastrointestinal disease diagnosis.

Cases in which predominant pathogens were identified in the gut microbiota and clinic diagnosis was reached accordingly

- Case 1.

**Figure 2:**
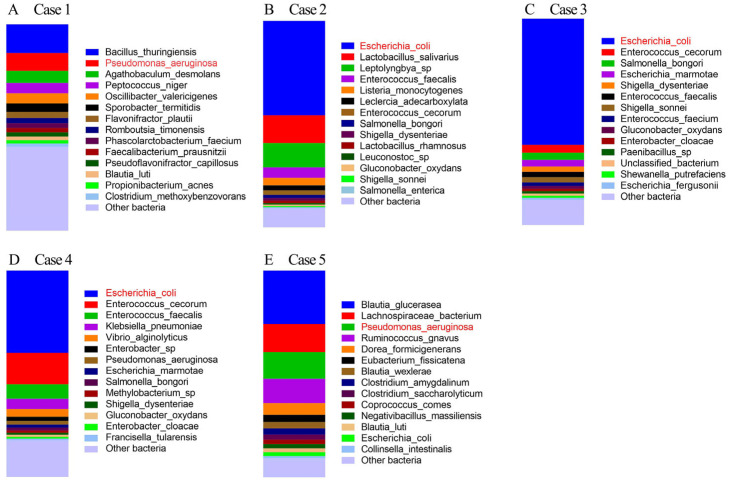
Gut microbiota composition of samples with obvious pathogenic bacteria infections. (**A**–**E**) High relative abundances of pathogenic bacteria identified in the gut microbiota of the five samples (Case 1 to Case 5). The diagnosed pathogens are marked in red.

Sick chickens from a medium-sized farm that housed around 25,000 chickens. The chickens exhibited symptoms including lethargy, lack of appetite, watery feces, intestinal mucosal hemorrhage, coronary hemorrhage, and cerebral congestion, with low mortality. While clinical signs and pathological autopsy did not yield a clear diagnosis, sequencing results identified *Pseudomonas aeruginosa* infection (Figure 2A). Targeted antibiotic treatment based on the sequencing results successfully cured the chickens and prevented further disease spread. These samples were also confirmed to be *P. aeruginosa* positive as revealed by pathogen cultivation results.

- Case 2.

Sick chickens from a medium-sized farm exhibited symptoms of pericarditis, perihepatitis, enteritis, and peritonitis, indicative of *E. coli* infection. Sequencing results confirmed the high abundance of *E. coli* in the gut microbiota (Figure 2B). The combined sequencing and autopsy findings led to the diagnosis of *E. coli* disease, and targeted antibiotic treatment was administered accordingly.

- Case 3.

Sick ducklings from a small-sized farm that housed around 1000 ducks. The sequencing results suggested *E. coli* infection (Figure 2C), which was confirmed by clinical signs observed during autopsy, consistent with *E. coli* disease in ducks.

- Case 4.

Sick piglets from a small-sized farm exhibited diarrhea. Sequencing identified high abundances of *E. coli* in the gut microbiota. Given that no *E. coli* vaccines were administered to these piglets, they were diagnosed with *E. coli* infection (Figure 2D). Pathological examination of duodenal tissue sections revealed significant morphological alterations, including inflammatory cell infiltration and thickening of the basal layer (Figure S1), indicative of a pathogen infection. The veterinarians developed a treatment plan based on these findings, including vaccination, improved feeding conditions, and enhanced environmental hygiene to prevent further outbreaks.

- Case 5.

An 8 year, 10-month-old male blue cat, presented symptoms of diarrhea and green stools with mild skin disease. Clinical diagnosis was difficult; however, sequencing identified a relatively rare *Pseudomonas aeruginosa* infection (Figure 2E). Following ciprofloxacin treatment, the cat recovered, and the owner reported improvements within one week.

Cases in which gut microbiota dysbiosis may be associated with inflammatory bowel disease (IBD)

- Case 6.

**Figure 3:**
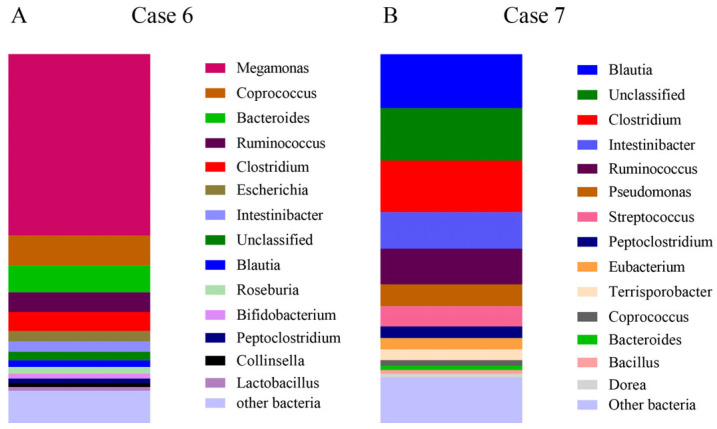
Gut microbiota composition of dog samples diagnosed with IBD. (**A**,**B**) The 15 most abundant bacterial genera identified in the gut microbiota of diseased animals Case 6 (**A**) and Case 7 (**B**).

A 10-year-old female teddy dog presented symptoms of diarrhea and sometimes blood in the stool. A colonoscopy revealed the occurrence of colonic ulceration, but the clinical diagnosis was inconclusive. Sequencing results indicated significant changes in the gut microbiota, including decreased abundances of *Clostridiales*, *Clostridium*, *Ruminococcaceae*, and *Faecalibacterium* compared with the healthy ones (Figure 3A and Table 1), indicative of IBD [34,35]. The observed phenomena are in accordance with the results reported by Suchodolski et al. [36]. Based on these findings, the dog was diagnosed with IBD, likely due to immune abnormalities caused by microbiota dysbiosis.

- Case 7.

A 12-year-old female Chinese field dog presented with diarrhea for one week, which was not relieved by a small dose of norfloxacin. Similarly to Case 6, clinical diagnosis was inconclusive, and sequencing revealed microbiota dysbiosis (Figure 3B and Table 1), supporting a diagnosis of IBD.

- Case 8.

**Figure 4:**
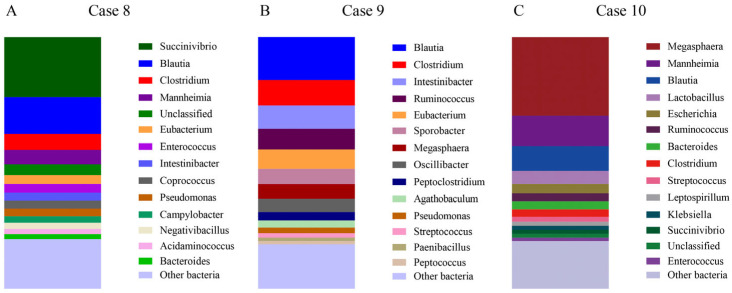
Gut microbiota composition of cat samples diagnosed with IBD. (**A**–**C**) The 15 most abundant bacterial genera identified in the gut microbiota of three diseased animals (Cases 8–10).

A young male blue cat, under one year of age, presented with diarrhea symptoms after changing diet approximately one week before coming to the pet clinic. On the day of the visit, the young cat exhibited symptoms of blood in the stool and vomiting. Common pathogens were ruled out by clinical tests, including a feline distemper test. Sequencing results indicated significant dysbiosis, with decreased abundances of *Bacteroides* and *Bifidobacterium* (Figure 4A and Table 2), leading to a diagnosis of IBD [36,37]. The observed phenomena are in accordance with the results reported by Marsilio et al. [38], which were confirmed by colonoscopy.

- Case 9.

A 6-year, 4-month-old male blue cat exhibited vomiting, diarrhea, and blood in the feces. The clinical diagnosis was inconclusive. Sequencing revealed a disturbance in the microbiota, consistent with characteristics of IBD, including a marked decrease in *Bacteroides* and *Bifidobacterium* abundance (Figure 4B and Table 2). A colonoscopy confirmed these findings, leading to a diagnosis of IBD.

- Case 10.

A 4-month-old female American shorthair cat presented with fever, vomiting, and diarrhea. Although initial treatment alleviated fever and vomiting, diarrhea persisted. Sequencing results confirmed a diagnosis of IBD (Figure 4C and Table 2), which was supported by clinical observations.

In cases of IBD in dogs and cats, similar treatment strategies were applied: immunosuppressive drugs for symptom control when severe or persistent, probiotics to regulate the gut microbiota, and a prescription hypoallergenic diet for dietary management. Additionally, animals were monitored every six months via ultrasound to assess intestinal wall thickness.

## 4. Discussion

Nanopore sequencing offers a convenient, relatively inexpensive, and real-time sequencing method with great potential for many applications, including point-of-care pathogen detection and microbiota profiling in humans [33,39,40,41]. However, its application in animal clinics remains limited. Accurate and rapid identification of the causes of gastrointestinal diseases is critical for the proper treatment of diseased animals. In this study, we developed a MinION full-length 16S rDNA sequencing analysis pipeline and evaluated its application potential in the diagnosis of animal gastrointestinal diseases. The results demonstrated that the pipeline could identify pathogens with high sensitivity and specificity within approximately 6 h, as demonstrated by the *Salmonella* contamination-based assay (Table S1). The turnaround time was significantly shorter than traditional cultivation-based methods, which typically require several days due to pathogen enrichment, cultivation, and subsequent assays [28]. Furthermore, the sequencing results facilitated the diagnosis of both pathogen infections and microbiota dysbiosis in animal gastrointestinal diseases, as evidenced by the case studies, highlighting its significant potential for clinical applications in veterinary medicine.

Among the cases where a relatively high abundance of pathogenic bacteria, which were identified through sequencing (Cases 1–5), Cases 1–4 were from the livestock and poultry outpatient clinic at the Yangzhou University Animal Hospital. These cases primarily originated from small and medium-sized farms, which often lack specialized management and veterinary expertise compared to larger farms [42,43]. When an animal epidemic occurs, a failure to rapidly and accurately diagnose the cause often leads to improper drug use, exacerbating economic losses, and hindering timely epidemic control. Additionally, the misuse of antibiotics can contribute to the spread of antimicrobial resistance [44,45,46,47]. Case 5, exhibiting typical symptoms of intestinal pathogenic infection that is common in pets, further exemplifies the challenges faced in companion animal diagnostics. Traditional diagnostic methods in companion animals are either time-consuming or limited in detecting a narrow range of pathogens, leaving rare or newly emerging pathogens undetected [48]. These limitations complicate pathogen detection and contribute to the improper use or misapplication of drugs in veterinary practice. In this study, we propose the use of MinION full-length 16S rDNA sequencing pipeline to rapidly and accurately diagnose animal diseases. This approach not only enables timely recovery of financial losses by farmers but also limits unnecessary antibiotic use, thus promoting animal welfare. For example, in Case 1, MinION technology enabled the rapid and accurate identification of *P. aeruginosa*, allowing the veterinarian to prescribe antibiotics with fewer side effects. Moreover, this method provides veterinarians with the ability to assess the risk of drug resistance in *P. aeruginosa*, facilitating treatment adjustments when quinolones are ineffective [49].

IBD is a common gastrointestinal disease in dogs and cats, often arising from multiple factors and challenging to diagnose [50]. The integration of clinical tools with knowledge of gut microbiota changes in both dogs and cats considerably shortens the time to diagnose IBD (Cases 6–10, Figure 3). Of note, without the identification of IBD, the patients with diarrhea may be inappropriately treated with antibiotics, which can further harm an already compromised gut microbiota [51,52]. Specific members in the healthy microbiota, such as those in the order *Clostridiales*, which play an important role in maintaining intestinal health in dogs [53], may recover more slowly after antibiotic disruption compared to other microbiota members [50,54]. Therefore, the developed MinION full-length 16S rDNA sequencing pipeline shows great promise in diagnosing IBD in dogs and cats and guiding effective treatment strategies for these companion animals.

In all evaluated cases, the entire process—from sample collection to pathogen and microbiota dysbiosis identification—was completed within approximately 6 h. Due to its compact design and user-friendly operation, MinION full-length 16S sequencing pipeline holds significant potential for application in veterinary clinics. However, our study has some limitations. The pipeline identifies potential pathogens using a database-dependent approach, which may fail to detect newly emerged pathogens that are not yet included in the database. Additionally, only five cases of pathogen infection and five cases of microbiota dysbiosis were analyzed in this study. More cases are needed to further evaluate the efficacy of the MinION full-length 16S rDNA sequencing analysis pipeline for diagnosing animal gastrointestinal diseases in future studies.

## Figures and Tables

**Table 1:** The relative abundance of IBD indicative taxa in Cases 6 and 7. The relative abundance of these bacterial groups in fecal samples from healthy and inflammatory bowel disease dogs [36] are also shown.

Bacterial Group	Healthy Medians % (min–max%)	Active IBD Medians % (min–max%)	Case 6	Case 7
*Clostridiales*	78.1 (21–97)	45.5 (1–94)	24.8	49.6
*Clostridium*	33.7 (5–84)	13.7 (0–82)	5.1	14.0
*Ruminococcaceae*	16.0 (0–46)	5.6 (0–54)	0	0.1
*Faecalibacterium*	0.1 (0–16)	0 (0–0)	0	0

**Table 2:** The relative abundance of IBD indicative taxa in Cases 8, 9, and 10. The relative abundance of these bacterial groups in fecal samples from healthy and inflammatory bowel disease cats [38] is also shown.

Bacterial Group	Healthy Medians % (min–max%)	Active IBD Medians % (min–max%)	Case 8	Case 9	Case 10
*Bacteroidales*	25.3 (1.2–56.4)	16.6 (0.7–55.3)	2.7	1.8	3.5
*Bifdobacterium*	1.2 (0–36.8)	0.2 (0.1–38.9)	0.3	0.1	0.3

## Data Availability

All sequences have been deposited in the China National GeneBank DataBase under Bioproject accession numbers CNP0003027.

## Supplementary Materials

The following supporting information can be downloaded at: https://www.mdpi.com/article/10.3390/microorganisms13040777/s1, Figure S1. Pathological sections of intestinal tissue from Case 4. Table S1. Detection thresholds for simulated contamination samples.

## Abbreviations

The following abbreviations are used in this manuscript:

IBD	Inflammatory Bowel Disease
SPF	Specific Pathogen Free
