# Supplementary material for: Development and Evaluation of a MinION Full-Length 16S rDNA Sequencing Analysis Pipeline for Rapid Diagnosis of Animal Gastrointestinal Diseases
Source: Microorganisms. 2025 Mar 28;13(4):777. doi: 10.3390/microorganisms13040777 (PMC12029435; doi:10.3390/microorganisms13040777)
Supplement: Supplementary file 1 [file microorganisms-13-00777-s001.zip › microorganisms-3508701-supplementary.pdf]

Table S1. Detection thresholds for simulated contamination samples.

Amount of *Salmonella* added in the fecal samples （CFU/g）	Population of bacteria in the fecal samples (16S copies/gram)	Relative abundance of *Salmonella* in the microbiota	Number of reads classified as *Salmonella*
0	6.588×10^{9}	0	0
0	6.588×10^{9}	0	0
0	6.588×10^{9}	0	0
1	6.588×10^{9}	1.06×10^{-9}	0
1	6.588×10^{9}	1.06×10^{-9}	0
1	6.588×10^{9}	1.06×10^{-9}	0
10	6.588×10^{9}	1.06×10^{-8}	0
10	6.588×10^{9}	1.06×10^{-8}	0
10	6.588×10^{9}	1.06×10^{-8}	0
100	6.588×10^{9}	1.06×10^{-7}	0
100	6.588×10^{9}	1.06×10^{-7}	2
100	6.588×10^{9}	1.06×10^{-7}	4

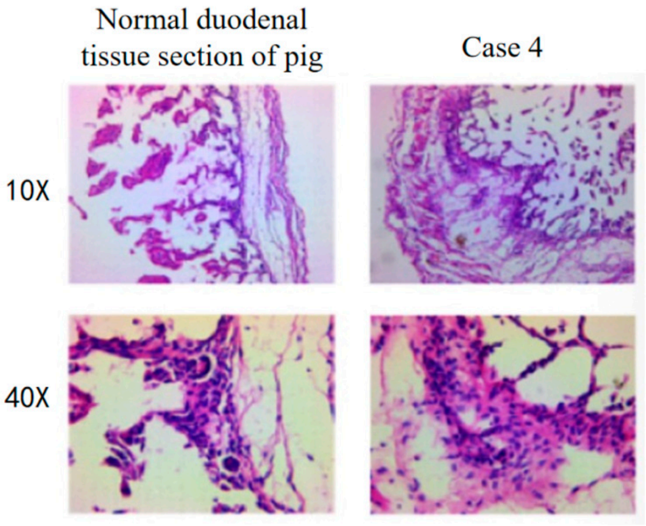

Figure S1. Pathological sections of intestinal tissue from Case 4
